# Nowcasting Sexually Transmitted Infections in Chicago: Predictive Modeling and Evaluation Study Using Google Trends

**DOI:** 10.2196/20588

**Published:** 2020-11-05

**Authors:** Amy Kristen Johnson, Runa Bhaumik, Irina Tabidze, Supriya D Mehta

**Affiliations:** 1 Ann & Robert H. Lurie Children's Hospital of Chicago Chicago, IL United States; 2 Northwestern University Chicago, IL United States; 3 School of Public Health University of Illinois at Chicago Chicago, IL United States; 4 Chicago Department of Public Health Chicago, IL United States

**Keywords:** health information technology, sexually transmitted infections, surveillance, infoveillance, infodemiology, Google Trends

## Abstract

**Background:**

Sexually transmitted infections (STIs) pose a significant public health challenge in the United States. Traditional surveillance systems are adversely affected by data quality issues, underreporting of cases, and reporting delays, resulting in missed prevention opportunities to respond to trends in disease prevalence. Search engine data can potentially facilitate an efficient and economical enhancement to surveillance reporting systems established for STIs.

**Objective:**

We aimed to develop and train a predictive model using reported STI case data from Chicago, Illinois, and to investigate the model’s predictive capacity, timeliness, and ability to target interventions to subpopulations using Google Trends data.

**Methods:**

Deidentified STI case data for chlamydia, gonorrhea, and primary and secondary syphilis from 2011-2017 were obtained from the Chicago Department of Public Health. The data set included race/ethnicity, age, and birth sex. Google Correlate was used to identify the top 100 correlated search terms with “STD symptoms,” and an autocrawler was established using Google Health Application Programming Interface to collect the search volume for each term. Elastic net regression was used to evaluate prediction accuracy, and cross-correlation analysis was used to identify timeliness of prediction. Subgroup elastic net regression analysis was performed for race, sex, and age.

**Results:**

For gonorrhea and chlamydia, actual and predicted STI values correlated moderately in 2011 (chlamydia: *r*=0.65; gonorrhea: *r*=0.72) but correlated highly (chlamydia: *r*=0.90; gonorrhea: *r*=0.94) from 2012 to 2017. However, for primary and secondary syphilis, the high correlation was observed only for 2012 (*r*=0.79), 2013 (*r*=0.77), 2016 (0.80), and 2017 (*r*=0.84), with 2011, 2014, and 2015 showing moderate correlations (*r*=0.55-0.70). Model performance was the most accurate (highest correlation and lowest mean absolute error) for gonorrhea. Subgroup analyses improved model fit across disease and year. Regression models using search terms selected from the cross-correlation analysis improved the prediction accuracy and timeliness across diseases and years.

**Conclusions:**

Integrating nowcasting with Google Trends in surveillance activities can potentially enhance the prediction and timeliness of outbreak detection and response as well as target interventions to subpopulations. Future studies should prospectively examine the utility of Google Trends applied to STI surveillance and response.

## Introduction

Gonorrhea, chlamydia, and syphilis continue to pose a significant public health challenge with approximately 3.7 million new diagnoses each year in the United States [1]. Rates of sexually transmitted infections (STIs) increased from 2017 to 2018, with gonorrhea, chlamydia, and syphilis showing a rise of 2.9%, 5.0%, and 14.9%, respectively [2]. Despite these documented increases, many cases remain undiagnosed and unreported; as a result, the true burden of these STIs is likely much greater [1]. The purpose of STI surveillance is to estimate the morbidity and mortality of disease as well as enhance the ability to predict and respond to disease patterns. Disparities in the rates of STIs by age, sex, race, and region have been observed; timely and accurate detection of these issues can support effective prevention and control [1].

National surveillance relies on mandatory laboratory and case reporting, a system that produces data that are often incomplete and limited in scope [3]. In addition to data quality concerns, underreporting and reporting delays result in missed opportunities to identify and respond to trends in disease and limit the ability to guide STI control [3,4]. Although web–based and electronic systems for laboratory and provider reporting can increase response timeliness, public health agencies must still apply the processes of matching and merging information, and detecting and removing duplicate cases and reports, which adversely impacts the timeliness of disease trend analysis [4]. Thus, there is a need to modernize and enhance surveillance systems to detect the burden of disease and improve the targeting of prevention and control activities [2].

Although the internet is not a “new” technology, it is relatively new to the surveillance of infectious diseases. In 2004, Eysenbach coined the term “infodemiology” to describe the distribution of determinants of information on the internet and “infoveillance” to refer to syndromic surveillance of disease via the internet [5]. As the internet is a portal for free, asynchronous, and anonymously available health information, search engine data may provide an additional venue for surveillance efforts, thus leading to earlier detection of trends and increased ability to monitor impacts and regional geographic spread. Previous studies have examined the utility of Google Trends to monitor infectious diseases such as influenza, dengue, Lyme disease, and COVID-19 [6-9]. Search engine data have the potential to provide efficient and economical enhancement to the  surveillance system established for STIs. Google Trends allows the download of deidentified search engine data trends, which can be used to investigate the implications of trends in STI-related search terms in relation to STI rates as well as facilitate disease nowcasting [10]. The term “nowcasting” refers to developing estimates of data in real time as the true data are being collected [11]. In reference to STIs, search engine data may provide the ability to determine trends in real time, which significantly enhances the current surveillance system [12]. This innovative tool has the potential to enable real–time surveillance of STIs, enhance understanding of changing STI trends, predict outbreaks, and increase the flexibility of the current system [3]. However, few studies, to date, have used Google Trends to predict or forecast disease. In a 2018 review, only 8.7% (9/104) of studies using Google Trends for infodemiology included predictions or forecasting [13].

We build on our previous study, which found that Google search trend volume was positively and statistically significantly correlated with reported annual rates of STIs at the state level [12]. In this study, we develop and train a predictive model using reported STI morbidity data from the Chicago Department of Public Health (CDPH), and we investigate the predictive capacity, timeliness, and ability to target interventions using Google Trends data. Cook County, primarily encompassing Chicago, is a large, diverse jurisdiction with the second highest case counts (second to LA County, California) in the country for chlamydia, gonorrhea, and primary and secondary syphilis [2]. In conjunction with this high disease burden, Chicago has high internet penetration [14], making it a suitable city for testing our model [15].

## Methods

A deidentified data set containing weekly STI case data for chlamydia, gonorrhea, and primary and secondary syphilis for 2011-2017 was obtained from the CDPH. The Institutional Review Board at the CDPH reviewed and approved the project proposal as exempt. Gonorrhea and primary and secondary syphilis have been nationally notifiable infections since 1944, and chlamydia, since 2010 [16]. The STI case data are aggregated to weekly counts for each case type (chlamydia, gonorrhea, and syphilis), with the date assigned based on the date the sample was obtained for testing. In addition to the STI diagnosis code, the data set includes race/ethnicity, age, and birth sex of the cases. Approximately 90% of all chlamydia and gonorrhea laboratory test results are reported via electronic laboratory reporting in real time via the Illinois National Electronic Disease Surveillance System. Data are deduplicated on a regular basis via built–in functionality or completed manually. All gonorrhea– and chlamydia–positive labs reported within >30 days are considered new cases. Reporting of syphilis cases is submitted electronically and managed manually. There were no specific STI city–wide prevention initiatives during the time period. The data are considered to be an accurate reflection of the rates of STIs in the city of Chicago [7].

The performance ability and predictive accuracy of the model rely on the selection of the search terms. To account for the breadth of terms that can possibly be used, the top 100 related search terms were obtained from Google Correlate using the initial term “std symptoms” [17]. All of the top 100 terms had correlation coefficients>0.85. After determining the related search terms, we established an autocrawler with Python [18] and used it to collect search volume data for each of the terms.

We used Google Health Application Programming Interface (API) (https://trends.google.com/trends/) data [19], which are a scaled proportion of the volume of all searches for all terms. The Google Health API results indicate the proportion of searches about the terms requested out of all searches that took place in Chicago per week for this time range, all multiplied by a consistent factor to increase ease of use. We excluded search terms with an insufficient search volume (ie, incomplete or absent search trend results returned by Google). The retrieved search trend data were averaged on a weekly basis. None of the search data in this study contain personal information or individualized records of Internet search history.

The distributions of each of the STI case counts by week comprised non–negative count variables. We applied Poisson regression modelling as dictated by the outcome distribution and in consideration of the Google Trends data [10]. The primary equation of the model is log(µ) = X_b_, where the response follows a Poisson distribution with parameters including mean μ. Coefficient vector b defines a linear combination X_b_ of the predictors X.

As the number of search query terms increases and exceeds the number of observations (in this case, the number of weeks), a curse-of-dimensionality and small-n–large-p affect the model. In addition, many of the query volumes may be zero because many queries are irrelevant (ie, assuming sparsity). Regularized regression schemes, such as lasso and elastic net, can solve this problem [11]. We used the elastic net penalty as it completes automatic variable selection and continuous shrinkage simultaneously, and it can select from a group of correlated variables, given the nature of correlated search queries [20].

We used a default parameter setting (10-fold cross validation for elastic net implementation in MATLAB 2017b to select the best regularization parameter lambda (λ) [21]*.* The queries selected for the best λ were used as the final set for the Poisson regression. We evaluated our approach for different values of ridge parameter *α*, starting from .5 to 1.0, and we chose the best parameter value based on the highest correlation coefficient between the predicted and actual STI counts.

To estimate the potential time advantage of using the internet-based search terms, we applied cross-correlation analysis. Search terms were filtered by applying cross-correlation analysis to estimate the temporal relationship between the STI cases and Internet search volume derived from each term. The results were obtained as product-moment correlations between the 2 time series. The advantage of using cross-correlation is that it accounts for time dependence between 2 time series variables. The time dependence between 2 variables is termed as lag, which indicates the degree and direction of associations. A lag of –1 or +1 for assessing correlation implies that the Google Trend data have shifted backward or forward by 1 week from the CDPH data. Cross-correlation analysis also reduces spurious correlations in the subsequent regression analysis by excluding irrelevant Internet search trends. Those search terms that lacked statistically significant correlations or a definite time lag or lead pattern were excluded. We measured our performance by using the following metrics between the predicted and actual STI counts: Pearson correlation *r* and mean absolute error (MAE).

## Results

### Epidemiologic Overview

From 2011 to 2017, there were 170,368 reported cases of chlamydia, 65,224 reported cases of gonorrhea and 4278 reported cases of syphilis (Table 1).

**Table 1:** Demographic characteristics and number of laboratory–confirmed reported cases of chlamydia, gonorrhea, and syphilis by year for Chicago, IL.

Characteristics				Chlamydia, n (%)		Gonorrhea, n (%)			Syphilis, n (%)	
Median age (years)				22		23			31	
**Sex, n (%)**										
	Male				66512 (33.63)			36282 (55.74)		2426 (74.39)
	Female				131255 (66.36)			28800 (44.26)		835 (25.61)
**Race, n (%)**										
		White			68687 (36.55)		20117 (31.82)			1965 (42.31)
		Black			107631 (57.27)		41003 (64.86)			2246 (48.36)
		Other			11594 (6.17)		2096 (3.31)			433 (9.32)
**Cases by year, n (%)**										
			2011		27686 (75.07)		8533 (23.14)			658 (1.70)
			2012		27729 (73.23)		9551 (25.224)			585 (1.50)
			2013		27325 (74.18)		8889 (24.23)			618 (1.60)
			2014		26990 (76.07)		7845 (22.11)			643 (1.80)
			2015		28256 (76.67)		7840 (21.27)			758 (2.05)
			2016		29776 (71.87)		10836 (26.15)			813 (1.96)
			2017		30292 (70.75)		11730 (27.40)			788 (1.84)

### Prediction

**Figure 1:**
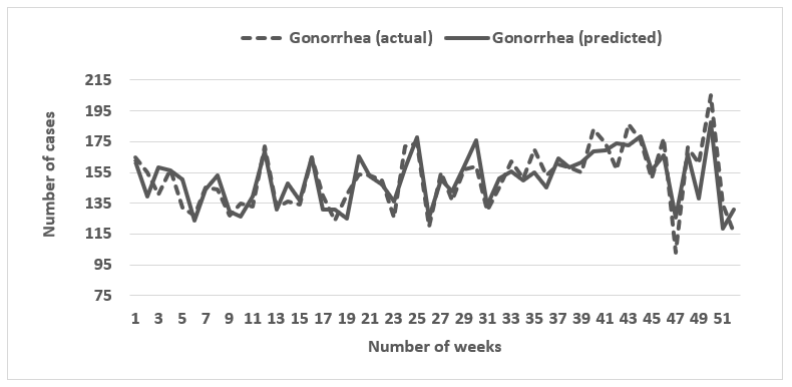
Graphical comparison between actual and predicted number of gonorrhea cases for 2017.

**Figure 2:**
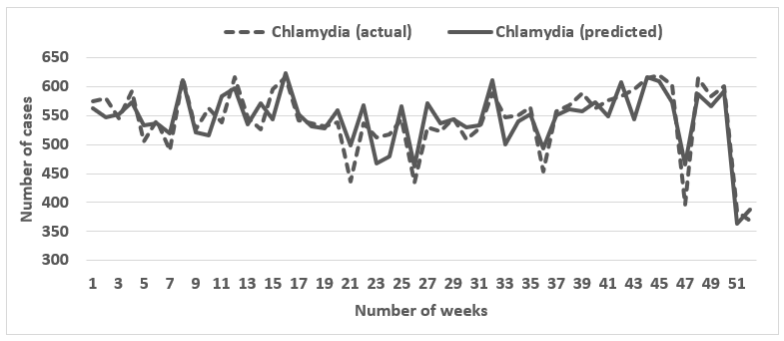
Graphical comparison between the actual and predicted number of chlamydia cases for 2017.

**Figure 3:**
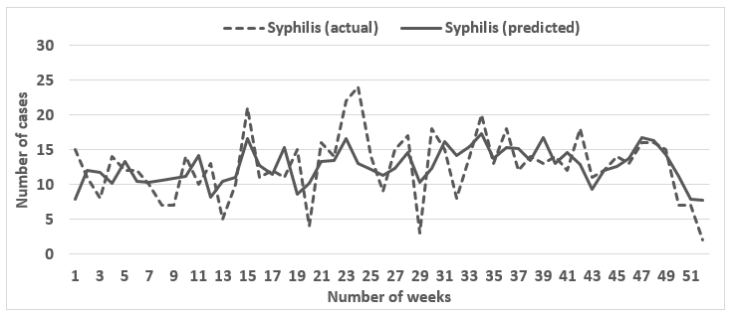
Graphical comparison between the actual and predicted number of syphilis cases for 2017.

We evaluated the predictions of STI cases from the search terms for 5 consecutive annual periods from 2011 to 2017 using elastic net regression. Table 2 enumerates the performance results for the model for years 2011 to 2017. For gonorrhea and chlamydia, actual and predicted STI values correlated moderately in 2011 (chlamydia: *r*=0.65; gonorrhea: *r*=0.72) but correlated highly (chlamydia: *r*=0.85-0.94; gonorrhea: *r*=0.82-0.90) from 2012 to 2017. However, for primary and secondary syphilis, the high correlation was observed only for 2012 (0.79), 2013 (0.77), 2016 (0.80), and 2017 (0.84), with 2011, 2014, and 2015 showing moderate correlations (0.55-0.70). Though all the Pearson correlation coefficients were significant, MAE ranged from 1.55%-3.02% for primary and secondary syphilis, 7.95%-19.04% for gonorrhea, and 17.04%-37.98% for chlamydia. Considering the high correlation in conjunction with the low MAE, the model performed the best for gonorrhea. Figures 1-3 present the graphical comparisons between the predicted and actual STI values for the year 2017 for gonorrhea, chlamydia, and syphilis. The search terms that appeared most frequently across all 3 diseases were “std symptoms in men,” “gonorrhea in men,” “yellow discharge,” “white creamy discharge,” “week pregnant,” and “white discharge.” All of the most common search terms relate to STI terminology, symptomology, or pregnancy (indicator of exposure to STI via condomless sex).

**Table 2:** Model prediction performance. *P*<.001 for all *r* values.

Year	Gonorrhea			Chlamydia			Primary and secondary syphilis		
	*r*	MAE^a^ (%)		*r*	MAE (%)		*r*	MAE (%)	
2011	0.72	12.56		0.65	36.12		0.70	2.50	
2012	0.86		11.56	0.85		25.34	0.79		1.55
2013	0.88	19.04		0.94	37.98		0.77	2.24	
2014	0.82	10.28		0.92	20.01		0.56	2.27	
2015	0.85	8.27		0.87	23.27		0.55	3.02	
2016	0.89	7.95		0.93	17.04		0.70	2.45	
2017	0.90	10.23		0.91	22.26		0.79	1.94	

^a^MAE: Mean absolute error.

### Subgroup Analyses

Following the same elastic net regression procedures, we developed separate models for the race (Black vs Nonblack), sex (male vs female), and age (<30 vs ≥30) subgroups. All the subgroup models across all years and all diseases (gonorrhea, chlamydia, and syphilis) performed optimally, showing high correlation values and low MAEs (see Multimedia Appendix 1). To illustrate performance, Tables 3-5 provide the results of elastic net regression for subgroup analyses for race, sex, and age for the gonorrhea case data for each year from 2011 to 2017. The subgroup models showed either similar or better performance than the full models across diseases and years: the correlations were high across subgroups of race, gender, and age (0.82-0.98), while the MAEs were low (2.67%-11.54%). The most frequent search terms for all 3 STIs for the category “Black” were “chlamydia treatment,” “signs of STD,” “smelly discharge,” “can pregnant,” and “creamy white discharge.”

**Table 3:** Subgroup (race) prediction performance for gonorrhea. *P*<.001 for all *r* values.

Year	Black		Nonblack		
	*r*	MAE^a^ (%)		*r*	MAE (%)
2011	0.89	6.41		0.92	2.67
2012	0.85	8.81		0.89	3.70
2013	0.92	11.54		0.93	5.15
2014	0.82	6.22		0.85	6.56
2015	0.90	4.54		0.84	5.86
2016	0.84	5.49		0.93	1.94
2017	0.92	4.6		0.91	2.71

^a^MAE: Mean absolute error.

**Table 4:** Subgroup (gender) prediction performance for gonorrhea. *P*<.001 for all *r* values.

Year	Male		Female		
	*r*	MAE^a^ (%)		*r*	MAE (%)
2011	0.88	4.73		0.92	2.67
2012	0.93	4.0		0.89	3.70
2013	0.96	5.25		0.93	5.15
2014	0.94	4.26		0.85	6.56
2015	0.93	3.94		0.84	5.86
2016	0.92	5.70		0.88	4.12
2017	0.91	6.13		0.90	3.92

^a^MAE: Mean absolute error.

**Table 5:** Subgroup (age) prediction performance for gonorrhea. *P*<.001 for all *r* values.

Year	Less than 30 years		30 years and above	
	*r*	MAE^a^ (%)	*r*	MAE (%)
2011	0.83	9.15	0.81	3.17
2012	0.91	7.67	0.81	3.13
2013	0.97	7.59	0.86	4.42
2014	0.90	7.25	0.80	2.87
2015	0.98	2.30	0.87	2.78
2016	0.98	1.98	0.91	3.56
2017	0.91	7.22	0.83	3.92

^a^MAE: Mean absolute error.

### Cross-Correlation Analysis

First, we conducted a cross-correlation analysis to identify the temporal relationship between the STI data and search terms (ie, a lag or lead pattern). Table 6 shows the results for chlamydia in the year 2017. The remaining results of the cross-correlation analysis on the search terms for each disease from 2012 to 2017 are included in Multimedia Appendix 2. Trends for the search terms “feel pregnant” (*r*=–0.28, *P*=.04), “treatment for chlamydia” (*r*=0.35, *P*=.01), “std” (*r*=–0.33, *P*=.02), “two weeks” (*r*=0.28, *P*=.04), “crabs std” (*r*=–0.36, *P*=.02), and “bleeding after period” (*r*=–0.32) coincided with the gonorrhea data in 2015. Weekly case counts of gonorrhea were preceded by 1 week by the trends of the following search terms: “does chlamydia” (*r*=–0.34, *P*=.01), “std symptoms in women” (*r*=0.31, *P*=.02), “gonorrhea” (*r*=–0.28, *P*=.04), and “after period” (*r*=–0.31, *P*=.02). The trends of the following search terms were correlated with the case counts of gonorrhea 1 week later: “wine while pregnant” (*r*=0.29, *P*=.04) and “talk to women” (*r*=–0.30, *P*=.03).

**Table 6:** Cross-correlation coefficients of reported cases of chlamydia using search term trend data for 2017^a^.

Search terms		Lags (week)		
		–1	0	1
**Internet search terms that preceded gonorrhea case counts by 1 week**				
	does chlamydia	–0.34^b^ (*P*=.01)	0.01	0.001
	std symptoms in women	–0.31^b^ (*P*=.02)	–0.04	0.16
	gonorrhea	–0.28^b^ (*P*=.04)	0.07	0.11
	samuel l jackson movies	0.29^b^ (*P*=.04)	–0.18	0.04
	after period	–0.31^b^ (*P*=.02)	0.09	-0.04
**Internet search terms that coincided with gonorrhea case counts**				
	treatment for chlamydia	0.00	–0.35^b^ (*P*=.01)	0.06
	a black eye	–0.11	–0.28^b^ (*P*=.04)	0.12
	std	0.09	0.33^b^ (*P*=.02)	0.12
	two weeks	–0.04	0.28^b^ (*P*=.04)	0.08
	crabs std	0.03	–0.36^b^ (*P*=.01)	0.003
	feel pregnant	–0.12	–0.28^b^ (*P*=.04)	–0.15
	bleeding after period	–0.09	–0.32^b^ (*P*=.02)	–0.005
**Internet search terms that lagged gonorrhea case counts by 1 week**				
	wine while pregnant	–0.10	–0.24	0.29^b^ (*P*=.04)
	talk to women	–0.06	0.09	–0.30^b^ (*P*=.03)

^a^Only significant cross-correlation coefficient values are shown in this table.

^b^Values indicate the maximum cross-correlation coefficient.

A separate regression analysis including only those search terms that coincided with and preceded the STI data by 2 weeks (ie, based on the results of the cross-correlation analysis in Table 7) was also conducted for recent years. The correlations between the actual and predicted cases of gonorrhea, chlamydia, and primary and secondary syphilis case counts are shown in Table 7.

**Table 7:** Correlations between actual and predicted cases of STIs for 2015-2017. *P*<.001 for all *r* values.

Year	Gonorrhea		Chlamydia		Primary and secondary syphilis	
	*r*	MAE^a^ (%)	*r*	MAE (%)	*r*	MAE (%)
2015	0.60	12.35	0.66	32.22	0.59	2.94
2016	0.67	13.97	0.77	28.75	0.57	2.95
2017	0.46	21.49	0.65	37.98	0.52	2.71

^a^MAE: mean absolute error.

## Discussion

### Findings

We performed a series of analyses to determine the predictive ability, timeliness, and performance of the Google Trends subgroups for the STI cases. The models performed consistently well overall across all diseases and time periods, showing moderate-to-high predictive power and low-to-moderate error. Applied nowcasting does not need to perform perfectly but must be reliable and consistent to inform disease control and response. As illustrated by the analyses of the Google Trends for influenza surveillance, the predictive performance of search volume may vary by disease, location, and over time [22,23]. However, variability is to be expected, and uniform success is not necessary for application in a surveillance setting. Google Flu Trends operated from 2008 to August 2015 and showed varying accuracy for predicting real–time outbreaks of influenza using Google search terms [24]. Google Flu Trends demonstrated that internet–based search term surveillance should not be used as a standalone surveillance system due to the existence of temporal and geographic variability; however, traditional surveillance systems could benefit by incorporating internet search term query data [24].

Our models were able to nowcast within a 1-week time frame, a substantial improvement from the delays observed when using traditional STI surveillance data. Further work is needed to determine thresholds for response, including determining what level of increase in case volume indicates a public health response and to what intensity. For example, a jurisdiction may decide that 10%, 20%, and 30% increases in search trend volumes may trigger a low intensity response (eg, provider awareness), public awareness, and active screening campaigns, respectively. Each of these thresholds and response activities need to be refined by local health departments based on epidemiologic trends and health department resources, but given the opportunity for real-time surveillance, and thus timely decision making and response provision, these efforts become urgent.

The ability to target prevention and control efforts to impacted subgroups is of great utility for public health efforts. Our model subgroup analyses performed better than or as efficiently as the aggregate models, demonstrating the ability to monitor trends in subgroups. These analyses were limited to the data available from our local health department; future studies should be conducted to refine and enhance subgroup performance. For example, control techniques may be influenced by outbreaks in specific neighborhoods; therefore, determining models fit for geographical subgroups (eg, community area and zip code) would be beneficial. Further, the analyses were conducted on retrospective data and involved using final cleaned surveillance data sets; future studies should be conducted prospectively in real time.

The search terms that most strongly correlated with the case counts for all 3 diseases were “std symptoms,” “gonorrhea in men,” “yellow discharge,” “white creamy discharge,” “week pregnant,” “yellow discharge,” and “white discharge.” All of these terms appear to be related to STI symptoms and are likely to be generated by those exposed to STIs (or cases). In a previous study, we established that those exposed to STIs are likely generating symptom-related search terms; we compared 2 different sexually active populations and found that compared to the student sample, a greater proportion of the clinical sample used the term “STD symptoms” or conducted symptom-related searches (47% vs 17%, *P*<.01) [17].

Google Trends supports credibility and transparency because these data are openly available, and our analyses are replicable by other investigators (see Multimedia Appendix 3). Further, search volume data access via Google Trends has remained continuously available since 2008 [10]. This study only used 1 source of open data; future studies could incorporate multiple sources of open source data to determine if data triangulation improves performance.

An evaluation of 8 state-wide health systems in North Carolina compared International Classification of Diseases-9 codes to a broad range of reported cases of notifiable communicable diseases, and showed that completeness of reporting ranged from 0% to 82% depending on the particular disease [25]. Thus a heightened degree of underreporting may lead to increasing error when using a tool such as Google Trends to predict disease. Audits of diagnoses would be necessary to estimate the amount of underreporting and potentially account for this issue in analyses. We did not find audits of STI underreporting in the published literature; based on our experience in STI surveillance, we estimate that of the 3 STIs under surveillance, syphilis may be the least likely to be underreported due to greater awareness among providers, given the severity of its clinical presentation (eg, lesions, generalized body rash, and neurosyphilis) and relatively infrequency.

### Limitations

The results of this study must be interpreted with the following limitations in mind. This study used STI case data from 1 jurisdiction in the United States; thus, it is unknown how nowcasting will function in other jurisdictions with different disease trends and search trend volumes. Future studies should include a representative selection of jurisdictions with high disease burdens and internet penetration. Our subgroup analysis was limited by the characteristics included in the STI case data available from the health department and did not include indicators such as zip code, community area, and socioeconomic status; therefore, we were not able to determine the impact of an analysis with these characteristics on target resources. Finally, our study used Google API, which is currently limited by Google to approved research institutions only, thus limiting the replication of the results to those who have API access.

### Conclusion

This is the first study to examine the utility of Google Trends search volumes to predict STI cases at a city level. Future studies should replicate procedures for other US jurisdictions and prospectively examine model performance while developing tolerance levels for false positives. Integrating nowcasting with Google Trends in surveillance activities can potentially enhance the timeliness of outbreak detection and response.

## Appendix

Multimedia Appendix 1 Subgroup analysis.

Multimedia Appendix 2 MATLAB code.

Multimedia Appendix 3 R code.

## Abbreviations

API: application programming interface
CDPH: Chicago Department of Public Health
MAE: mean absolute error
STI: sexually transmitted infection
