# Supplementary material for: Nowcasting Sexually Transmitted Infections in Chicago: Predictive Modeling and Evaluation Study Using Google Trends
Source: JMIR Public Health Surveill. 2020 Nov 5;6(4):e20588. doi: 10.2196/20588 (PMC7677015; doi:10.2196/20588)
Supplement: Multimedia Appendix 1 [file publichealth_v6i4e20588_app1.docx]

Supplemental tables

Subgroup (race) prediction performance for chlamydia

|  | **Black** | | **Non Black** | |
| --- | --- | --- | --- | --- |
| YEAR | r | MAE | r | MAE |
| 2011 | .87  (p<.0001) | 18.45 | .85  (p<.0001) | 11.37 |
| 2012 | .82  (p<.0001) | 18.69 | .87  (p<.0001) | 11.03 |
| 2013 | .90  (p<.0001) | 29.24 | .85  (p<.0001) | 30.85 |
| 2014 | .87  (p<.0001) | 15.30 | .86  (p<.0001) | 14.82 |
| 2015 | .86  (p<.0001) | 15.26 | .86  (p<.0001) | 15.26 |
| 2016 | .88 | 12.10 | .91 | 4.02 |
| 2017 | .90 | 12.43 | .88 | 6.51 |

Subgroup (gender) prediction performance for chlamydia

|  | **Male** | | **Female** | |
| --- | --- | --- | --- | --- |
| YEAR | r | MAE | r | MAE |
| 2011 | .79  (p<.0001) | 10.97 | .88  (p<.0001) | 18.50 |
| 2012 | .76  (p<.0001) | 9.99 | .88  (p<.0001) | 18.40 |
| 2013 | .82  (p<.0001) | 18.70 | .94  (p<.0001) | 13.93 |
| 2014 | .87  (p<.0001) | 10.38 | .86  (p<.0001) | 15.77 |
| 2015 | .84  (p<.0001) | 9.64 | .84  (p<.0001) | 5.86 |
| 2016 | .88 | 8.26 | .92 | 12.61 |
| 2017 | .90 | 9.33 | .93 | 11.74 |

Subgroup (age) prediction performance for chlamydia

|  | **Less than 30** | | **30 and above** | |
| --- | --- | --- | --- | --- |
| YEAR | r | MAE | r | MAE |
| 2011 | .93  (p<.0001) | 16.82 | .80  (p<.0001) | 5.37 |
| 2012 | .97  (p<.0001) | 9.96 | .79  (p<.0001) | 4.64 |
| 2013 | .98  (p<.0001) | 18.26 | .91  (p<.0001) | 6.84 |
| 2014 | .92  (p<.0001) | 18.71 | .79  (p<.0001) | 6.22 |
| 2015 | .99  (p<.0001) | 5.87 | .83  (p<.0001) | 5.56 |
| 2016 | .97  (p<.0001) | 8.38 | .81  (p<.0001) | 6.55 |
| 2017 | .94  (p<.0001) | 11.89 | .85  (p<.0001) | 5.86 |

Subgroup (race) prediction performance for Syphilis

|  | **Black** | | **Non Black** | |
| --- | --- | --- | --- | --- |
| YEAR | r | MAE | r | MAE |
| 2011 | .94  (p<.0001) | .95 | .78  (p<.0001) | .88 |
| 2012 | .86  (p<.0001) | .95 | .84  (p<.0001) | .87 |
| 2013 | .85  (p<.0001) | 1.26 | .84  (p<.0001) | .98 |
| 2014 | .81  (p<.0001) | 1.21 | .87  (p<.0001) | .92 |
| 2015 | .84  (p<.0001) | 1.23 | .89  (p<.0001) | .94 |
| 2016 | .83  (p<.0001) | 1.29 | .79  (p<.0001) | 1.24 |
| 2017 | .80 | 1.08 | .76 | 1.22 |

Subgroup (gender) prediction performance for Syphilis

|  | **Male** | | **Female** | |
| --- | --- | --- | --- | --- |
| YEAR | r | MAE | r | MAE |
| 2011 | .92  (p<.0001) | 1.29 | .79  (p<.0001) | .53 |
| 2012 | .89  (p<.0001) | 1.06 | .88  (p<.0001) | .41 |
| 2013 | .87  (p<.0001) | 1.60 | .94  (p<.0001) | 1..93 |
| 2014 | .74  (p<.0001) | .59 | .88  (p<.0001) | 1.29 |
| 2015 | .83  (p<.0001) | 2.24 | .82  (p<.0001) | .46 |
| 2016 | .71 | 2.23 | .71 | .51 |
| 2017 | .80 | 2.02 | .78 | .48 |

Subgroup (age) prediction performance for Syphilis

|  | **Less than 30** | | **30 and above** | |
| --- | --- | --- | --- | --- |
| YEAR | r | MAE | r | MAE |
| 2011 | .83  (p<.0001) | 1.41 | .75  (p<.0001) | 1.98 |
| 2012 | .84  (p<.0001) | 1.04 | .81  (p<.0001) | 1.12 |
| 2013 | .89  (p<.0001) | 1.04 | .85  (p<.0001) | 1.25 |
| 2014 | .60  (p<.0001) | 1.59 | .76  (p<.0001) | 1.38 |
| 2015 | .89  (p<.0001) | .97 | .85  (p<.0001) | 1.31 |
| 2016 | .83  (p<.0001) | 1.05 | .82  (p<.0001) | 1.49 |
| 2017 | .74  (p<.0001) | 1.09 | .82  (p<.0001) | 1.55 |

**Top ten search terms correlated with diseases**

| **Gonorrhea** | **Chlamydia** | **P&S Syphilis** |
| --- | --- | --- |
| discharge | discharge | feelings meme |
| can chlamydia | can chlamydia | can chlamydia |
| after period | hpv symptoms | home std test |
| yellow discharge | chlamydia | discharge |
| feelings meme | gonorrhea in men | in two weeks |
| hpv symptoms | gonorrhea | light blood |
| bleeding after period | after period | does chlamydia |
| gonorrhea in men | feelings meme | eddie murphy movies |
| white discharge | credit score good | denzel washington movies |
| why am i spotting | chlamydia in women | white creamy discharge |
|  |  |  |
